# New Appliances and Things Medical

**Published:** 1900-06-23

**Authors:** 


### NEW APPLIANCES AND THINGS MEDICAL.

[We shall be glad to receive, at our Office, 28 & 29, Southampton Street, Strand, London, W.C., from the manufacturers, specimens of all new preparations and appliances which may be brought out from time to time.]

### WHITE'S PATENT ASEPTIC WATER PILLOW.

(Albert Browne, 3, Bowling Green Street, Leicester.)

In the construction of this water pillow the idea has been to separate the fabric from the indiarubber. In the ordinary pillow the foundation, if it may be so called, is of canvas, this being rendered watertight by being enclosed in layers of a rubber compound. One of the evils of this mode of construction is that if the water gets to the canvas it spreads by capillary attraction, and, separating the rubber from the fabric, spoils the pillow, and renders it very difficult to repair. By making a canvas bag into which a thin pliable pure rubber bag is inserted, much as the inner tube is inserted into a pneumatic tyre or a bladder into a football, the makers of this pillow gain the advantages of being able to deal separately with the two substances of which it is made—the protective fabric and thin elastic bag within—the result being that the whole thing can be sterilised after use by immersion in boiling water, and that if the indiarubber water chamber should be torn or punctured it can easily be patched like a bicycle tyre. It seems a good idea. Certainly the sample we have examined forms a very soft and pliable pillow.

